# Health concerns of veterans with high-level lower extremity amputations

**DOI:** 10.1186/s40779-018-0183-4

**Published:** 2018-10-26

**Authors:** Elahe Faraji, Mostafa Allami, Nafiseh Feizollahi, Amir Karimi, Amir Yavari, Mohammadreza Soroush, Majid Moudi

**Affiliations:** Janbazan Medical and Engineering Research Center (JMERC), NO.17, Farrokh St., Moghaddas Ardebily Ave., Chamran Highway, Tehran, Iran

**Keywords:** War- related trauma, High level lower extremity amputation, Health needs assessment

## Abstract

**Background:**

The aim of the study was to identify health concerns of veterans with high-level lower extremity amputations.

**Methods:**

Through a cross-sectional study, general practitioners, an orthopedic specialist, psychologists, psychiatrists, physiotherapist and prosthetists examined 100 veterans using a short-form health-related quality of life questionnaire (SF-36) that assessed their ability to perform activities of daily living (ADL), instrumental activities of daily living (IADL) and life satisfaction (SWLS) after hip disarticulation or hemi-pelvectomy amputations. The assessment tool was designed to gather statistically useful information about their health needs.

**Results:**

The means of the Physical Component Summary (PCS), Mental Component Summary (MCS), SWLS, ADL and IADL were 48.58 ± 29.6, 33.33 ± 22.0, 19.30 ± 7.7, 48.10 ± 10.5 and 5.08 ± 1.8, respectively. Somatization, depression, and anxiety were the most prevalent disorders; among the veterans who were visited by psychiatrists, 11.6% had a history of hospitalization in a psychiatry section, and 53.2% had a psychiatric visit. Regardless of their injury in battle, 34% of veterans were hospitalized. Hearing problems were common, and about four-fifths of the participants suffered from at least one orthopedic condition. Neuroma (49%) was the most common stump-related complication during orthopedic evaluations, though the prevalence of phantom pain was 81% during the pain assessment. A total of 87% of the participants had a history of wearing a prosthesis, but only 29% wore a prosthesis at the time of the present study. The Canadian-type of prosthesis was uncomfortable and not useful (27%) and excessively heavy (10%) according to the amputees.

**Conclusions:**

Understanding veterans’ characteristics and special needs are important to make sure that enough facilities and services are afforded to them. These findings emphasize the importance of paying close attention to different dimensions of health in veterans and can help health providers identify health needs and make regular assessments**.**

## Background

Health is a concept emphasizing social and personal resources, as well as physical capacities. It is defined as being in a good physical, mental and social state, and it is not limited to a lack of illness or disability [1]. Organized assessments are required to identify health problems and to develop health care plans in special groups [2]. Agha et al. [3] stated there is a large difference in the health status and subsequent resource use between veterans and the general patient population. They concluded that comparisons of veteran care with non-veteran care need to consider these differences, and health care planning and resource allocation to veterans should not be based on data extrapolated from non-veteran patient populations.

Battlefield extremity injuries such as amputation cause the majority of long-term disabilities [4]. People with amputations have special physical, mental, and social conditions during their lifetime. This situation is more severe in military veterans and may affect their response to treatments [5]. War-related high-level lower extremity amputations, such as hip disarticulation and trans-pelvic amputations, are complicated. In the previous decades, nearly half of the patients who had hemi-pelvic amputations would pass away, but today, considering advancements in surgery and anesthesia, this figure has decreased to 0–10% [6–10]. This population is more at risk for health problems.

Daily living activities among veterans with hip disarticulation and trans-pelvic amputation have been evaluated. The most affected activity was ascending and descending the stairs, and approximately 83% of veterans were limited in at least one domain of daily living activities [11]. These amputees were better in the mental domain than the physical domain, and complaints of pain in the stump occurred in approximately 33% of veterans. The research concluded that veterans with high-level lower extremity amputations would need life-long care [12].

The aim of this study was to identify health *concerns of veterans with* high-level lower extremity amputations (short transfemoral, hip disarticulation and hemipelvectomy) and to determine their health priorities. Knowing the long-term complications and interventions in the field of treatment and rehabilitation may improve and/or prevent the progression of these conditions and increase the individual’s independence.

## Methods

The descriptive, cross-sectional study was conducted between Jun 21, 2016 and Mar 16, 2017. All veterans with unilateral short transfemoral hip disarticulation or hemipelvectomy from Iran were listed by the Veterans and Martyrs Affairs Foundation (VMAF), and 205 veterans were invited to participate in a health needs assessment study at a recreational campground. The inclusion criterion was willingness to take part in the study, and the exclusion criterion was incomplete data collection. In Iran, a veteran is defined as a person who has participated in a war, suffers from war-related physical and/or mental injuries, and has been diagnosed with a certain degree of disability and injury according to the VMAF [13]. A team was constituted to determine required health professionals and facilities at different stages of the project. Based on the results of the previous study on this group and the literature, a short list of health priorities was defined to assess health conditions. Before the beginning of the assessment, a group consensus was reached about the project proposal. The assessment team consisted of general physicians, an orthopedic specialist, psychiatrists, psychologists, physiotherapists and prosthetists. The following tools were used:

1. The health-related quality of life assessed by the SF-36 questionnaire. The short-form health survey is a self-reporting set of generic, coherent, and easily administered quality of life measures that is utilized by managed care organizations for routine monitoring and assessment of care outcomes in adult patients. It has been found that the Persian version of the questionnaire has an acceptable reliability (α = 0.77–0.90) [14].
2. Global cognitive judgments of satisfaction with one’s life were measured using the satisfaction with life scale (SWLS). The SWLS does not assess satisfaction with life domains, but it allows subjects to integrate and weight these domains in whatever way they choose. The validity and reliability of the Persian version of the questionnaire was previously determined (α = 0.83 and test-retest reliability coefficient = 0.69) [15].
3. The Barthel index of activities of daily living (ADL) and instrumental activities of daily living (IADL) were used, which represent key life tasks that people need to be fully independent. ADLs are the basic self-care tasks, but IADLs require more complex thinking skills, including organizational skills. The Persian version was found to be consistent, valid and recommended for research projects (α and intraclass correlation coefficient (ICC) > 0.75) [16, 17].
4. The Symptom CheckList-90-Revised (SCL-90-R) questionnaire was used as a brief self-reported psychometric instrument [18]. The Persian version of the SCL-90-R instrument (α = 0.78–0.89) was used in order to screen psychological problems and symptoms of psychopathology in the participant [19]. The Diagnostic and Statistical Manual of Mental Disorders (DSM-IV) is a standardized classification system that was used by the psychiatrists for the diagnosis of mental health disorders [20].
5. An orthopedic specialist, physiotherapists and prosthetists completed musculoskeletal and prosthesis and orthosis use forms. The content validity of the forms was verified by JMERC’s approved specialists. The Numeric Pain Rating Scale (NPRS) was applied as a unidimensional measure of chronic pain intensity and to assess the worst level of pain experienced [21]. The Modified Oswestry Disability questionnaire (MODQ), as a gold standard of low back functional outcome tools, was used to measure a patient’s permanent functional disability of low back pain [22, 23]. The Trinity Amputation and Prosthesis Experience Scales (TAPES) is a multidimensional assessment of adaptation to amputation and prosthesis use [24]. In this study, the Persian version of the MODQ, with Cronbach’s α of 0.69, was applied [25]. The Persian version of TAPES was available with a Cronbach’s alpha of 0.70 [26]. If there was a need for more accurate evaluation and/or interventions, referral to a related physician specialist and/or service provider was recorded.
6. Physicians completed a comprehensive patient assessment form that included the medical history and physical examination. If there was a need for more accurate evaluation, referral to a related specialist was recorded. The form is used generally in JMERC’s health needs assessment projects for veterans with different kinds of impairments. The content validity of the form was verified by JMERC’s approved specialists. The mean, standard deviation, frequency and percent of the collected data were calculated using SPSS 16 software (14.0, SPSS Inc., Chicago, IL, USA).

## Results

### Demographic

Of the 205 veterans invited, 100 participated in the study (response rate: 48.8%). Approximately 97% of participants were men aged between 35 and 65 years with a mean of 51.51 ± 5.5 years. Approximately 99% of them were married; however, 77.7% of them were single before being injured. Approximately 2% had an academic education at the time of injury versus 29% at the time of the study. Approximately 14% suffered from chemical warfare injuries. and 25% suffered from psychiatric disorders. Approximately 96% were amputated during a traumatic war event and/ or in initial interventions. Shrapnel was the cause of 80% of the injuries. A mean of 31.68 ± 2.7 years has passed since the injury.

### Quality of life and satisfaction with life

Altogether, 31% of the participants assessed their health conditions as being good, very good or excellent. The means for PCS and MCS were 33.33 ± 22.0 and 48.58 ± 29.6, respectively. More details are provided in Table 1. The mean score for quality of life was 40.95 ± 25.1. The mean score for satisfaction with life as a whole was 19.30 ± 7.7. Just 6% of the participants were extremely satisfied.

**Table 1:** Quality of life and the satisfaction with life sub-scales

Quality of life sub-scales	Mean ± SD
Physical function (PF)	42.35 ± 28.3
Role physical (RP)	28.50 ± 38.7
Body pain (BP)	33.72 ± 26.5
Vitality (V)	52.83 ± 30.0
Mental health (MH)	53.93 ± 27.7
General health (GH)	28.75 ± 22.8
Social function (SF)	54.25 ± 32.5
Role emotion (RE)	33.33 ± 44.1

### ADLs, IADLs and sport activities

The mean score of the participants’ ADL (out of 100) was 48.10 ± 10.5. The mean score for IADL (out of 8) was 5.08 ± 1.8. Ten percent of patients had the highest possible score, and 2% had the lowest score. A total of 82% could independently dress, and 28% were dependent for bathing. Bowel and bladder control were independent in 92% and 80% of the participants, respectively. Using the toilet was not independent in 11% of participants, and 83% were independent for transfer (bed to chair and back). A total of 81% has independent mobility on level surfaces, though only 61% has independent mobility when ascending and descending the stairs. In instrumental ADLs (IADLs), 25% could not go shopping at all, 27% were totally independent in preparing food and could alone prepare food, 21% did not take part in any household activities, 30% all laundry was done by others, 48% could travel independently on public transportation, 67% had total independence in taking their medicine, and 70% were totally able in perform financial activities. A total of 53% of the participants had participated in sport activities for an average of 6.08 ± 5.1 h per week. Swimming (23%) and sitting volleyball (21%) were among the activities.

### Mental health

Results from SCL-90 revealed that body complaints, depression, and anxiety had high prevalence among the participants (details are provided in Table 2). The mean of the Global Severity Index (GSI) was 1.30 ± .81. A total of 77 participants were visited by psychiatrists. Approximately 11.6% and 53.2% had a history of hospitalizations in psychiatric wards and psychiatric visits, respectively. According to the DSM-IV, in axis I, there was cognitive disorder in 1.2%, substance dependence in 1.2%, mood disorder in 12.9%, anxiety disorder in 54.5%, and somatoform disorder in 1.2%. In axis II, there was 3.8% of participants with personality disorder. GAF (Global Assessment of Functioning Scale) scores of 9.0%, 11.6%, 18.1%, 19.4%, 19.4%, 18.1% and 3.8% for participants were 31–40, 41–50, 51–60, 61–70, 71–80, 81–90 and 91–100, respectively, in axis V. Anxiety disorder was the most prevalent disorder, followed by mood disorders.

**Table 2:** Mean and standard deviation of SCL-90-R subscales

Type of the psychological dimensions	< 2 sick	1–2 suspicious	< 1 healthy	Mean ± SD
Somatization	42	30	29	1.64 ± 0.9
Anxiety	31	28	41	1.35 ± 0.9
Obsessive compulsion	27	35	38	1.38 ± 0.9
Hostility	25	32	43	1.29 ± 1.0
Paranoid ideation	20	51	29	1.34 ± 0.8
Interpersonal sensitivity	18	31	50	1.16 ± 0.9
Depression	32	26	42	1.42 ± 1.0
Phobic anxiety	10	33	57	0.90 ± 0.8
Psychoticism	10	28	62	0.81 ± 0.7

### Neuromusculoskeletal conditions

The type of surgical technique used for amputation was myoplasty in 41% and myodesis in 17% of patients. A total of 8% needed stump surgery again, based on the opinion of the orthopedic surgeon. A total of 3 participants had upper limb amputation. A total of 87% of the participants suffered from at least one orthopedic complication. Regarding the number of surgeries done, 61% of the cases had 3 or more surgeries. The most common problem of the stump was neuroma (49%) (Table 3). The prevalence of phantom pain was 81%, and the prevalence of stump pain was 88%. Based on the amputees’ pain prioritization, the first priority was pelvic pain (40%) and, in order of priority, the next was knee pain (28%), hand, wrist and low back pain (15%) and low back pain (14%). A total of 52% of subjects had one or more pain at the time of study. There was low back pain in 80% of patients, and the mean modified Oswestery disability index was 59.97 ± 17.2. Disability due to low back pain in 28% of patients was severe and in 34% was crippling. A total of 87% of the participants had a history of wearing a prosthesis, but 29% wore a prosthesis at the time of the study. The patients stopped wearing the prosthesis if it was uncomfortable and not useful (27%) or excessively heavy (10%). Scores of the psychosocial adjustment subscales, satisfaction with prosthesis subscales, and the activity restriction subscales are presented in Table 4. Two common types of assistive technology used at home were the crutch (92%) and wheelchair (36%). These assistive devices were used outside and inside the home at a rate of 86% and 30%, respectively.

**Table 3:** Stump complications and other orthopedic disorders

Categories	Complications	*n* (%)
Stump complications	Contain redundant soft tissue	13 (13%)
	Too thin stump	8 (8%)
	Myositis ossificans	16 (16%)
	Skin adhesion	12 (12%)
	Skin grafting	15 (15%)
	Neuroma	49 (49%)
	Skin scar or ulcer	4 (4%)
	Inflammation	9 (9%)
	Discharge of pus	5 (5%)
	Osteomyelitis	3 (3%)
	Hip subluxation	4 (4%)
Other orthopedic disorders	Lumbar surgery	6 (6%)
	Osteoporosis	6 (6%)
	Contralateral ankle and foot disorders	65 (65%)
	Contralateral knee disorders	80 (80%)
	Contralateral hip disorders	32 (32%)
	Upper limb disorders	52 (52%)

**Table 4:** Scores of trinity amputation and prosthesis experience subscales (*N* = 29)

Scales	Content	Range	Mean ± SD
Satisfaction with prosthesis subscales	Aesthetic satisfaction	4–20	13.96 ± 3.2
	Weight satisfaction	1–5	2.32 ± 1.2
	Functional satisfaction	5–25	14.39 ± 4.7
Activity restriction subscales	Social restriction	0–8	2.82 ± 2.4
	Functional restriction	0–8	3.50 ± 2.4
	Athletic activity restriction	0–8	6.07 ± 1.9
Psychosocial adjustment subscales	Adjustment to limitation	5–25	12.17 ± 5.1
	Social adjustment	5–25	19.27 ± 4.9
	General adjustment	5–25	15.75 ± 4.1

Spinal orthoses (55%), knee support (33%), orthopedic shoes (32%) and upper-extremity orthoses (49%) were prescribed for the patients. The most commonly prescribed spinal orthoses was the soft lumbosacral orthosis (LSO), and the most commonly prescribed walking aids and mobility assistive device were crutches (41%) and a standard wheelchair (26%). Nearly half of the patients (47%) needed a shower wheelchair. From the prosthetist’s point of view, it was not possible to make a prosthesis for 9% of the participants, although wearing a prosthesis was recommended for 57% of patients. Additionally, 3% needed a prosthesis for an upper limb amputation.

### General physical health

A total of 15% of participants said that they had been hospitalized due to their amputation during the last year, though 34% of them were hospitalized regardless of the amputation. The mean body mass index (BMI) of the patients was 29.01 ± 5.1, indicating that the patients were overweight. *The mean systolic* blood pressure was 127.71 ± 20.1, and the mean diastolic blood pressure was 75.76 ± 14.5. Thirty-three percent of patients had a history of hypertension. Referrals to otolaryngologists and urologists were common. A total of 21% of the patients were current smokers. Table 5 contains a summary of physical diseases/problems or disorders.

**Table 5:** Descriptive statistics of physical diseases/problems or disorders

Type of diseases	History (%)	Recommendation to visit a specialist (%)
Urinary diseases	37	28
Digestive diseases	50	11
Cardiovascular diseases	44	20
Eye problems	11	11
Hearing problems	66	33
Neuropsychiatric disorders	36	21
Respiratory and lung diseases	18	10
Infectious diseases	11	2
Dermal diseases	12	11
Endocrine diseases	17	10
Sexual disorders	50	38

## Discussion

The present health assessment recognized and described the characteristics of a veteran population with high-level lower extremity amputation (short transfemoral, hip disarticulation and hemipelvectomy). The health needs of this population were identified.

Quality of life is important in investigating the short- and long-term consequences of amputation [27–29]. Numerous studies were done regarding the quality of life in amputee veterans. In this study, the quality of life of the group was lower both for males and females in physical and mental dimensions compared to the normal population [30], which is similar to previous studies. Houdek [6] evaluated 5 hemipelvectomy amputees using advanced prostheses. The score of the physical subscale (but not the psychological subscale) was lower compared to the normal population [6]. Studying the quality of life of 605 lower-extremity amputees who were 18 years old and above, it was shown that MCS and SF-36 scores were significantly lower in the participants when compared to the normal population [28]. Reiber et al. [31] studied 298 soldiers who were in the Vietnam War (mean age: 60.7 years) and 283 soldiers from the Iraq War (mean age: 29.3 years). According to SF-36, 70.7% of the Vietnam War soldiers and 85.5% of the Iraq War soldiers had good to excellent health status despite having severe injuries [31]. Dougherty et al. [32] compared 11 amputee soldiers from the Iraq War and 13 amputee soldiers from the Vietnam War. Sixty-nine percent of the Iraq war soldiers and 73% of those who were present in the Vietnam War reported a good or very good to excellent quality of life, and the results showed that the quality of life was similar in both groups. In this study, the high-level lower extremity amputees rated their health status lower than the two previous studies, which may be related to several factors. In a systematic review article, Christensen et al. [27] stated that the level of physical activity, level of amputation, back pain, years of education, and the intensity and duration of phantom pain could have an effect on the quality of life in lower extremity amputee veterans, while the level of physical activity could have a positive effect on quality of life [27]. In another study, employment status, using assistive device, prosthetic wearing, secondary complications, phantom pain, and stump pain were shown to be important predictors of MCS and PCS scores [28]. Furtado et al. [33] insisted that pain and physical function have a significant effect on quality of life. These factors must be considered to ensure the reintegration of veterans into the society and to maintain quality of life. Quality of life is considered an important tool in monitoring rehabilitation program outcomes and is generally used in order to compare the effectiveness of different interventions. Many amputees live long after the war, and this highlights the importance of their quality of life, including affective, physical and social dimensions of life.

The mean score of the participants’ ADL and IADL were approximately 50%, although no participant was totally dependent in ADL and IADL. DʼAlleyrand et al. [34] studied the side effects of traumatic hemipelvectomy after 2 years of follow-up and claimed that out of 13 war injuries that led to hemipelvectomy amputation, only 2 patients retained normal bowel and bladder control, and the other patients needed 44 additional surgeries. Evaluating the ADL of veterans with bilateral lower limb amputations showed that the highest level of help was needed for transportation (27.8%) [35]; however, this was more common in short transfemoral, hip disarticulation and hemipelvectomy amputees. In this study, 54% participated in sporting activities, although 50% of the bilateral lower limb amputees participated in sporting activities [36]. It was suggested that physical activity should be incorporated into the rehabilitation programs for these patients [37, 38]; however, it is important to consider contextual factors, such as the appropriate urban environment, which can cause differences in ADL and IADL.

In the present study, the mean score of satisfaction with life was 19.6, which was between extreme satisfaction and extreme dissatisfaction. Physical and mental problems and limitations in various individual and social factors may be effective in lowering the level of satisfaction with life [39]. The level of satisfaction with life has been reported to be lower in people with amputation compared to the normal population [40, 41]. In fact, side effects and secondary complications of amputation can be influential for decreasing the level of satisfaction with life. The participants who were satisfied and those who were dissatisfied were equal in number, and we recommend further investigation of the potential causative factors.

Psychological disorders have been identified in veterans that experienced numerous physical problems [42, 43]. The epidemiology of psychological disorders reveals that more than half of the veterans with short transfemoral, hip disarticulation and hemipelvectomy had been in outpatient care and more than one-tenth had a history of hospitalization, which was greater than that found in a study that evaluated 103 veterans with bilateral upper limb amputation [44]. A total of 3% of those patients had a history of hospitalization in psychiatric wards. There is the need for further investigation of the factors affecting mental and psychological health, the prevalence of different disorders, and the required help and support for military personnel with physical impairment [41]. In the present study, there was post-traumatic stress disorder in about one-third of the participants; however, anxiety disorder was more frequent than depression. It is important to provide these patients with enough care and support to face the related psychological challenges.

Regarding anxiety and depression, the prevalence was similar. It is worth mentioning that long-term stress and anxiety make the patient vulnerable to depression. War survivors, because of their high levels of post-traumatic stress disorder experiences, may have an increased overlap between anxiety disorder and mood disorder [45]. In addition, symptoms of somatoform disorder are more obvious when the patients do not have the chance to talk about their psychological problems [46]. The psychiatrists’ prescribed treatment plan changed to 41.1%, and hospitalization occurred for 1.2% of patients. The results revealed that the highest level of dissatisfaction was with physical and functional limitations, followed by emotional and vitality dissatisfaction. It may be concluded that veterans who suffer from physical and functional limitations are dependent on others and have lower satisfaction with life.

Midterm and long-term observations have revealed that more than half of the patients who lost a limb due to a trauma need a detailed diagnosis of mental and psychological problems [47–52]. Post-traumatic stress, anxiety, depression, and substance abuse are among the most common disorders in these patients [53]. Post-traumatic stress disorder occurs in approximately two-thirds of lower-extremity amputees and is the most prevalent cause of psychological problems in these patients [31, 54, 55]. Depression and anxiety disorders and substance abuse affect about a quarter and 6% of the amputee patients, respectively [31, 52, 54, 56]. Two hundred and ninety-eight soldiers from the Vietnam (mean age: 60.7 years) War and 283 soldiers from the Iraq War (mean age: 29.3 years) have been studied for psychological disorders. The prevalence of depression (24.5%, 24%) and post-traumatic stress disorder (37.6%, 58.7%) was observed to be high among the participants [31]. These problems also reduce the patients’ ability to cope with their physical disability [57]. Although anxiety and depression are prevalent in war survivors, the widespread range of physical problems in these patients establishes the hypothesis of the presence of somatoform symptoms, especially in patients who have decreased chance of expressing their problems.

A significant percent of amputees suffer from long-term chronic pain (50–80%) [31, 58]. In the present study, the prevalence of low back, phantom, and stump pains in the participants was higher than the prevalence in similar studies. Veterans from the Vietnam War and Iraq War were reported to suffer from phantom pain (72.2%, 76.0%), chronic low back pain (36.2%, 42.1%), and stump pain (48.3%, 62.9%) [31]. In another study, phantom pain and stump pain were observed in 78.8% and 51% of the participants, respectively. A significant relationship was also observed between stump and phantom pain [59]. Buchheit et al. [60] stated that 64.5% of the amputees reported a clinical pain score higher than 3 in a week, 61% experienced pain in the residual limb, and 58% had a phantom pain. Gallagher et al. [61] showed that after amputation, approximately 48% of the patients experienced stump pain and 69% had phantom pain. Smith et al. [62] studied pain and sensitivity in 92 unilateral lower limb amputees for a month and found that 71% suffered from low back pain.

The diagnosis of the source of chronic pain is helpful [63]. A significant relationship was observed between stump and phantom pain [59, 64], which indicates the importance of having a multidimensional view of pain. Stump pain is associated with other medical situations and low levels of adaptability to the disability, while phantom pain is related to aging, gender, above knee amputation, pre-amputation support, medical complications, and being content with the esthetic of the prosthesis [61]. Back pain was reported to be more annoying than phantom and stump pain [62, 65].

Incorrect posture and compensatory movement have been proposed to be the most important cause of low back pain. Fatigue during activities and factors related to the prosthetic device that cause inappropriate movements could alter the loading pattern on the spine and can lead to the development of back pain [66–68]. In the current study, more than half of these patients were prescribed a soft lumbosacral orthosis, which was due to the presence of low back pain. These finding indicate that although the veterans used walking aids for their mobility, the walking puts great pressure on the spine.

Chronic pain is a very common cause of inability in traumatic amputations [61, 69], though the etiology of chronic pain is not acknowledged completely [70] and investigations have shown that psychological factors may have a role in the transition to chronic pain [71]. Pain can limit and deteriorate functionality, physical activity, professional performance, and psychological status of these patients [72, 73]. Furtado et al. [33] stated that pain and physical functionality were very important in the quality of life. Self-management strategies in the form of maintaining physical fitness and support from health specialists could be beneficial in pain management and preventing the pain from turning into chronic pain [74, 75]. Amputees still suffer from chronic pains despite the medical advances. Therefore, deeper studies with the approach of new therapeutic and rehabilitation interventions and applying the new technologies, and paying more attention to the comprehensive management programs and other non-invasive interventions to prevent, treatment and rehabilitation of the chronic pains is necessary.

Regaining functional independence is the most important aim of rehabilitation programs in lower limb amputees [76, 77]. Wearing prosthesis following a hemipelvectomy improves individual’s balance and mobility, muscular tone and strength**,** expands cardiovascular health and increases functional mobility [6]. Prosthetic rehabilitation has an important role in mobility and returning the patients to their social lives and involves numerous complications. A total of 71% of the participants did not use prosthesis at the time of the study despite the fact that only 9% participants could not use prosthesis at the same time due to their stump and health conditions. Prosthesis wearing percentage in amputees with major traumatic limb loss from the Vietnam war and the Iraq and Afghanistan wars were 78.2% and 90.5%, respectively [31] although, in study of Fernández et al. [78] have been reported that 34.7% of Canadian prosthesis user continued to use their prosthesis in an 8-year follow up and that the time of prosthetic wear was 12.5 h a day. Kralovec et al. [79] studied 43 patients with hip disarticulation or hemipelvectomy for 10 years (2000–2010), and they reported that 18 (43%) were wearing their prosthesis successfully. The amputees with successful prosthesis fitting were wearing the prosthesis for an average of 5.8 h a day, with one or two walking aids [79].

Age, gender, cause and level of amputation, and general health status play important roles in wearing prosthesis [80, 81]. Due to high levels of energy expenditure in the amputees, having high levels of motivation leads to increased importance of prosthetic use [82–87]. Losing a limb shifts the weight to the other side of the body, and the amputee would need more muscular strength and endurance on that side to use a prosthesis. In addition, the loss of joints and mobility limitations puts more pressure on other parts of the body [88–90]. The main goals of a successful prosthetic rehabilitation program are being comfortable and having a functional and visually beautiful prosthetic [6, 91–93]. However, some studies indicate that gender, age and etiology are not among the determining factors in wearing a prosthesis [78]. Ludwigs et al. [94] stated that choosing the proper parts when designing a prosthesis for an amputee could have large effects on mobility and individual independence in patients after hip disarticulation or hemipelvectomy [94]. Fernández et al. [78] stated that amputees wearing Canadian prosthesis felt that factors such as socket intolerance, walking problems, high energy expenditure, and moving among other people could cause rejection of wearing a prothesis [78]. In Kralovec et al.’s study [79], the only limiting factor was coronary artery disease. Body mass index, other medical conditions, and demographic characteristics were not significantly related to the success rate of prosthesis fitting. The results from this study indicated that successful rehabilitation is possible in these patients, and an increase in the BMI, age, depression, or any other medical problems should not reduce the motivation for prosthesis use. Many of the patients who wore a prosthetic limb enjoyed their lives more and used their prosthesis for long hours during the day [79]. Considering that a Canadian-type prosthesis is heavy and difficult to use, many users with high level lower extremity amputations use walking aids and/or wheelchairs as assistive technologies to allow them to walk in an easier and a more accessible way, although secondary complications of these mobility methods may arise with the passage of time, thus decreasing their mobility [95]. These studies are very much in-line with our results showing that the reasons of ceasing to wear the prosthesis were: a) the prosthesis was uncomfortable and inefficient, b) they preferred to walk with walking aids or use a wheelchair because of the heaviness of the prosthesis, and c) uncomfortable socket fit. Based on the participants’ opinions, prosthetists and prosthetic facilities are not informed and prepared to offer prosthetic rehabilitation services to people with high level lower extremity amputations.

The factors affecting prosthesis abandonment in this group are complicated. Successful prosthetic rehabilitation of high level lower extremity amputees who abandoned prosthesis use, especially in people who are accustomed to other modes of ambulation, were due to the high cost, the use of a team-based approach, accurate and multi-dimensional evaluation, and ensuring the implementation of the program. It is suggested that, along with age-related changes in the amputees, more investigation is needed in order to evaluate prosthetics and physical functionality. Although the prosthesis replaces the missing limb, many limitations remain. It is also important to consider secondary issues while evaluating the health status of the veterans.

A 24-year follow-up study has shown that veterans with lower-extremity amputations had higher mortality rates than the normal population (21.9% vs. 12.1%) [96]. In this study, the most prevalent illnesses were: kidney and urinary tract diseases (37%), digestive diseases (50%), cardiovascular diseases (44%), eye diseases and tinnitus (43%), hearing loss (41%), neurological disorders (36%), sexual disorders (50%), and diabetic conditions (15%). Cruz et al. [97] investigated 229 retired veterans with below or above knee amputations in hospitals from 1994 to 2001. Most of them suffered from other complications that increased their mortality rate. The most common cause of mortality was cardiorespiratory complications [97]. In 298 soldiers from the Vietnam War (mean age: 60.7 years) and 283 soldiers from the Iraq War (mean age: 29.3 years), the presence of dermal problems due to prosthesis use were 51.05% and 58.0%, the percentage with hearing loss was 47.0% and 47.0%, and the percentage with brain injuries was 3.4% and 33.9%, respectively [31]. Previous studies have indicated that the sound of a firearm and the explosion of mines and bombs during the war may be responsible for one-third of the hearing problems in exposed people, and hearing loss was observed in people who worked in military garrisons [98–100]. Cruz et al. [97] stated that their participants had a history of long term hospitalization (average 15 days, minimum 3 days, and maximum 105 days) which**,** in addition to high financial expenses, resulted in a decrease in the functionality of the patients as well.

## Conclusions

Understanding veterans’ characteristics and special needs is important in order to make sure that enough facilities and services are being afforded to them. The findings from this research emphasize the importance of paying close attention to different dimensions of health in veterans and can help health providers to identify health needs and make regular assessments. In addition to physical limitations due to amputation and prosthetic rehabilitation, other injuries and their side effects could be closely related to quality of life. This knowledge suggests that future longitudinal and case control studies should compare the health status in veterans that have different levels of amputation. In the long term, the knowledge will help to improve the quality of life and wellbeing of amputees.

## Abbreviations

ADL: Activities of daily living
DSM-IV: The Diagnostic and Statistical Manual of Mental Disorders
IADL: Instrumental activities of daily living
JMERC: Janbazan Medical and Engineering Research Center
MCS: Mental Component Summary
MODQ: Modified Oswestry Disability questionnaire
NPRS: Numeric Pain Rating Scale
PCS: Physical Component Summary
SCL-90-R: Symptom CheckList-90-Revised
SF-36: Short Form health survey
SWLS: Satisfaction with life scale
TAPES: Trinity Amputation and Prosthesis Experience Scales
VMAF: Veterans and Martyr Affair Foundation
